# Usability of Immortalized Porcine Kidney Macrophage Cultures for the Isolation of ASFV without Affecting Virulence

**DOI:** 10.3390/v14081794

**Published:** 2022-08-16

**Authors:** Ken-ichiro Kameyama, Tomoya Kitamura, Kota Okadera, Mitsutaka Ikezawa, Kentaro Masujin, Takehiro Kokuho

**Affiliations:** Exotic Disease Group, National Institute of Animal Health (NIAH), National Agriculture and Food Research Organization (NARO), 6-20-1 Josuihoncho, Kodaira-shi, Tokyo 187-0022, Japan

**Keywords:** African swine fever virus, immortalized porcine kidney macrophage, viral isolation, virulence

## Abstract

Immortalized porcine kidney macrophage (IPKM) cells are highly susceptible to major African swine fever virus (ASFV) isolates. To clarify the compatibility of this cell line for ASFV isolation from biomaterials, animal experiments and *in vitro* isolation were performed. Pork products seized at international airports were subjected to virus inoculation in pigs (*in vivo*) and IPKM cell cultures (*in vitro*) to examine the viability and virulence of the contaminating viruses. Moreover, the viruses isolated using IPKM cells were inoculated into pigs to assess the virulence shift from the original materials. All pigs that were inoculated with either homogenate samples of seized pork product or IPKM-isolated ASFVs developed typical symptoms of ASF and died (or were euthanized) within the term of the animal experiments. The success rate of virus isolation in IPKM cells was comparable to that observed in porcine primary alveolar macrophage (PAM) cells. The IPKM cell line would be an ideal tool for the isolation and propagation of live ASFVs with high efficiency and enhanced usability, such as immortal, proliferative, and adhesive properties. The isolated viruses retained biologically similar characteristics to those of the original ones during isolation *in vitro*.

## 1. Introduction

The African swine fever virus (ASFV) is the causative agent of African swine fever (ASF), which is a contagious viral disease of domestic pigs and wild boars. ASFV is an enveloped, double-stranded DNA virus belonging to the genus *Asfivirus*, the family *Asfaviridae* [1]. Based on the phylogenetic tree analysis of the *B646L* gene, which encodes the structural p72 protein, ASFVs are currently divided into at least 24 genotypes [2]. Although all of these genotypes are originally present in sub-Saharan Africa, outbreaks with genotype I virus occurred in Europe, the Caribbean, and in South America between 1957 and the mid-1990s, and genotype II virus emerged in Georgia in 2007 and spread to Europe, the Asian-Pacific countries, and the Caribbean since then [3,4,5].

ASFV is strongly resistant to inactivation in the presence of organic materials, such as blood, meat, and feces [6,7]. Such strong resistance is an important characteristic that allows the virus to spread easily across borders beyond a long distance. It has been suspected that the swill from a cruise ship from the African continent is the possible source of the recent global epidemic of this disease, which has emerged since 2007 [8]. Thus, mass transportation could represent a great risk for the wider spread of the virus into naive countries. It has been reported that, in Japan, many foreign tourists bring pork meat and products illegally from ASFV-endemic areas that have been detected to be “ASFV positive” by PCR at border controls [9]. To conclude the infectivity and virulence of the viruses contaminating those illegally imported items, virus isolations, and animal experiments are routinely conducted at our laboratory.

Recently, we reported a newly established cell line of pig macrophage lineage, the immortalized porcine kidney macrophage (IPKM) cell line, which is sustainable and highly susceptible to both cell-culture-adapted and field isolates of ASFV [10,11]. The IPKM cell line supports virus replication in vitro as efficiently as do primary macrophages without introducing unexpected mutations in the viral genome. In this study, we attempted to apply this novel cell line to the direct isolation of ASFVs from contaminated pork products that were imported illegally from abroad and examined virulence in these cells using animal experiments in comparison with that of the viruses that contaminated the original materials. Together with the data obtained by next-generation sequencing (NGS) of the cell-culture-isolated viruses, we concluded that the IPKM cell line could be a favorable substitute for primary macrophages for the *in vitro* isolation of field viruses of ASFV without affecting their genetic and biological features.

## 2. Materials and Methods

### 2.1. Pork Products

Four different pork products, two (1–21 and 1–22) from China and two (20901-1 and 201202) from the Philippines, were seized independently by the Animal Quarantine Service (AQS), Ministry of Agriculture, Forestry and Fisheries (MAFF) at international airports in Japan from January 2019 to December 2020 (Table 1). All materials were checked for the presence of ASFV genes by quantitative PCR, then used for the experiments performed in this study.

### 2.2. Animal Experiment

Fourteen 8-week-old LWD pigs were used for two animal experiments in total. The animal experiment procedures were carried out in compliance with the regulations outlined in the Guide for the Care and Use of Laboratory Animals of the institution, the Guidelines for Proper Conduct of Animal Experiments of the Science Council of Japan [12], and the ARRIVE guidelines [13]. The animal study was reviewed and approved by the Institutional Animal Care and Use Committee (approval numbers 20-046, 20-075, and 20-082). A humane endpoint was considered to have been reached when animals exhibited markedly reduced activity and lay down, justifying euthanasia on welfare grounds. Pigs that survived throughout the experiment were euthanized by injection of xylazine and ketamine and then subjected to necropsy examination.

#### 2.2.1. Experiment 1

One milliliter each of a 10% suspension of pork products, 1–21, 1–22, or 20901-1 was inoculated intramuscularly into 3, 2, and 2 pigs, respectively. The body temperature and clinical symptoms of all 7 pigs were recorded daily, and whole-blood samples of the 2 pigs inoculated with 20901-1 were collected daily. Body temperature was measured in the rectum. The blood samples of the 5 pigs inoculated with 1–21 or 1–22 were collected at the start (Day 0) and end of the experiment period (Day 14) and on the days after those at which the body temperature exceeded 40 °C. Tissue specimens of the inoculated pigs (tonsil, lung, spleen, and kidney) were collected at the postmortem inspection and subjected to viral DNA detection.

#### 2.2.2. Experiment 2

One milliliter each of 10^2^ TCID_50_ of the AQS-C-1-22 or AQS-P-20901-1 isolate (described in detail in Section 2.5) was inoculated intramuscularly into 5 and 2 pigs, respectively. The body temperature of all pigs was recorded daily throughout the experimental period. Whole-blood samples were collected from each pig daily. Several organs were collected as described in Experiment 1.

All the experiments using ASFV were performed in the biosafety level 3 facility of the institution, which was accredited by the national authority of Japan.

### 2.3. Quantitative PCR (qPCR)

qPCR was performed according to the method of King [14], with minor modifications. Briefly, viral DNA of blood and 10% tissue homogenate samples was extracted using a High Pure Viral Nucleic Acid Kit (Roche, Basel, Switzerland), and amplification was performed under the following conditions: 50 °C for 2 min, 95 °C for 20 s, and 35 cycles of 95 °C for 3 s and 58 °C for 30 s using a 7500 fast real-time PCR system with the TaqMan Fast Advanced master mix (Thermo Fisher Scientific, Waltham, MA, USA).

### 2.4. Cell Cultures

IPKM cells were routinely maintained as described previously [10,11]. Briefly, the cells were cultured in Dulbecco’s Modified Eagle’s Medium (DMEM, Nacalai Tesque, Kyoto, Japan) supplemented with 10% fetal bovine serum (FBS), 10 µg/mL bovine insulin (Merck, Darmstadt, Germany), 25 µM monothioglycerol (Wako, Osaka, Japan), and antibiotics in cell culture plates and flasks (Sumitomo Bakelite, Tokyo, Japan). Porcine alveolar macrophage (PAM) cells were prepared from 8-week-old LWD pigs as described previously [10] and stored at −80 °C until use.

### 2.5. In Vitro Isolation of ASFV from Seized Pork Meat Products

The pork products were manually homogenized with a Micro Smash homogenizer (Tomy digital biology, Tokyo, Japan) and diluted in DMEM to prepare 10% or 20% (wt/vol) suspensions (Lot No.1). After centrifugation at 8000× *g* for 1 min, cleared supernatants were passed through a 0.45-µm sterile filter (Merck, Darmstadt, Germany), then diluted to give 2% and 0.2% suspensions. PAM (1 × 10^5^ cells/well) and IPKM (4 × 10^4^ cells/well) cells were seeded in each well of 96-well cell culture plates 1 day before the assay. Forty wells each of the culture plates were inoculated with 25 µL of 2% and 0.2% suspension of pork products and incubated at 37 °C in a 5% CO_2_, 95% air incubator in the presence of porcine RBCs. At 7-day intervals, 50 µL of the supernatant was serially passaged twice to freshly prepared PAM and IPKM cell cultures, as described above. The wells showing hemadsorption (HAD) were examined by microscopy. The viruses isolated by IPKM cells from pork products 1–21, 1–22, 20901-1, and 201202 were termed AQS-C-1-21, AQS-C-1-22, AQS-P-20901-1, and AQS-P-201202, respectively.

For verification of the reproducibility of IPKM versus PAM cells for virus isolation, new 10% homogenates of the pork products (Lot No.2) were prepared. These samples were inoculated to 3 sets of IPKM cells and 3 batches of PAM cells with the same method mentioned above. Every batch of PAM cells was collected from independent pigs.

### 2.6. Next-Generation Sequencing

ASFV isolates collected from the 3rd passaged supernatant in the IPKM and PAM cell cultures were used. The viral DNA of the isolated viruses was purified using the High Pure Viral Nucleic Acid Kit (Roche, Basel, Switzerland). The libraries of viral DNA were processed with the Ion Express Plus Fragment Library Kit for the AB Library Builder System (Thermo Fisher Scientific, Waltham, MA, USA). Whole-genome sequences of ASFV were determined by the Ion PGM system with the Ion PGM Hi-Q View OT2 Kit and Ion PGM Hi-Q View Sequencing Kit (Thermo Fisher Scientific, Waltham, MA, USA). All procedures were carried out according to the manufacturer’s instructions. The entire genome sequences of isolated viruses using IPKM cells were registered at DDBJ/EMBL/GenBank under accession numbers LC659086 (AQS-C-1-21), LC659087 (AQS-C-1-22), LC659088 (AQS-P-20901-1), and LC659089 (AQS-P-201202).

### 2.7. Phylogenetic Tree Analysis

A maximum likelihood tree based on the *B646L* gene sequences was generated with Tamura’s three-parameter model using MAFFT and MEGA version 7.0 software. The accession numbers of the sequences used in this tree are as follows: AQS-C-1-21 (LC659086), AQS-C-1-22 (LC659087), AQS-P-20901-1 (LC659088), AQS-P-201202 (LC659089), 47_Ss_2008 (KX354450), 26544_OG10 (KM102979), Lisboa60 (KM262844), Benin_97_1 (AM712239), E75 (FN557520), BA71V (NC_001659), NHV (KM262845), OurT_88_3 (AM712240), Mkuzi_1979 (AY261362), ASFV-SY18 (MH766894), Georgia07 (FR682468), Odintsovo02_14 (KP843857), Pig/HLJ/2018 (MK333180), Pretorisuskop96_4 (AY261363), Warmbaths (AY261365), Wart80 (AY261366), Tengani_62 (AY261364), Wuhan_2019-1 (MN393476), Malawi_lil-20_1 (AY261361), Ken05_Tk1 (KM111294), Kenya1950 (AY261360), Ken06_Bus (KM111295), RSA_2_2008 (MN336500), and ETH/5a (KT795361).

## 3. Results

### 3.1. Infectivity and Virulence of ASFVs in Illegally Imported Meat Products

**Figure 1:**
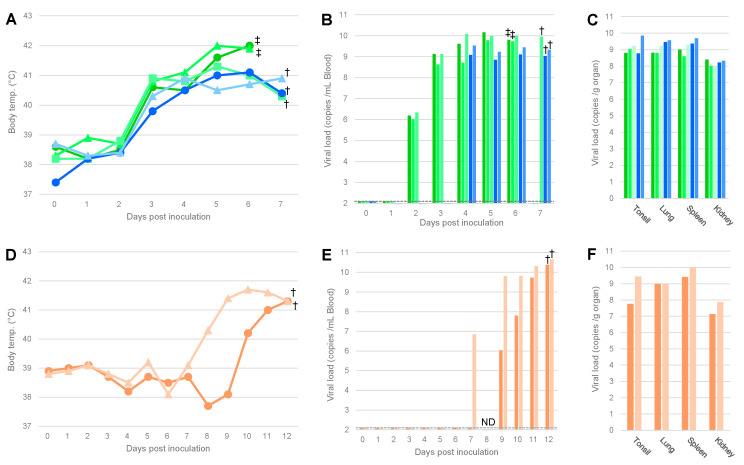
Body temperature and viral load of pigs inoculated with pork meat homogenates. The body temperature (**A**,**D**) and viral copy numbers in blood (**B**,**E**) and organs (**C**,**F**) of pigs inoculated with a 10% suspension of pork products are shown. Samples No. 1–21 (green) and 1–22 (blue) were from China (**A**–**C**), and sample No. 20901-1 (orange) was from the Philippines (**D**–**F**). The identification numbers of pigs are indicated by the shape of markers or sorting order of bars: Nos. 1, 2, and 3 are indicated by circles, triangles, and squares (**A**) or ordered from left to right (**B**,**C**), respectively. Each symbol means euthanasia by humane endpoint (†) or death (‡), and the broken lines in (**B**,**E**) indicate the detection limit (=100 copies/mL of blood). ND: not determined.

To examine whether the viruses contained in the illegally imported pork products were infectious to pigs, animal experiment 1 was conducted. In this experiment, 1 mL of a 10% suspension prepared from three different pork meat homogenates, 1–21, 1–22, and 20901-1, were inoculated into three, two, and two pigs, respectively. For 1–21 and 1–22, the body temperatures of all pigs increased (>40 °C) at 3 dpi. At 4 dpi, virus loads in the blood and serum increased in all pigs. Two out of the three pigs inoculated with the suspension of 1–21 died at 6 dpi, whereas the remaining pig was euthanized at 7 dpi. The two pigs inoculated with the suspension of 1–22 also reached the humane endpoint at 7 dpi. These results indicated that the viruses contained in samples 1–21 and 1–22 were infectious and highly virulent (Figure 1A–C). On the other hand, pyrexia was observed in the pigs inoculated with 20901-1 from 8 or 10 dpi, and viremia was detected from 7–9 dpi (Figure 1D,E). These pigs showed astasia and were euthanized at 12 dpi. The viral loads in the blood reached 10^10^ copies/mL, and those in the organs were also high (10^7^–10^10^ copies/g) (Figure 1E,F).

### 3.2. Isolation of ASFV from Pork Meat Products Using PAM and IPKM Cell Cultures

To isolate ASFV from pork meat products in vitro, 2% and 0.2% suspensions of the homogenates were inoculated into PAM and IPKM cells. After two rounds of serial passage, HAD reaction was confirmed in both cell cultures in the four independent samples, i.e., 1–21, 1–22, 20901-1, and 201202, that were subjected to the assay (Table 1). The number of HAD-positive wells increased in the second round of passage compared with the first, but not in further passages (Table 2). The viral loads of the 10% sausage homogenates (Lot No.1) of 1–21, 1–22, 20901-1, and 201202 quantified by qPCR were 6.58, 6.79, 4.13, and 7.68 log_10_ copies/mL, and the virus titers were <10^1.5^, 10^2.7^, <10^1.5^, and 10^2.5^ TCID_50_/mL, respectively.

For sample 1–21, HAD was confirmed in 28 and 4 wells in the second round of passage of PAM cells inoculated with the 2% and 0.2% suspension of the homogenate, respectively. Conversely, IPKM cells inoculated with both concentrations of the homogenate died on all 40 wells in the first round, and HAD reaction was confirmed in only 5 wells of IPKM cell cultures inoculated with the 2% suspension of the homogenate in the second round of passage. For sample 1–22, HAD reaction was confirmed in all wells of PAM and IPKM cells inoculated with the 2% suspension of homogenate, and in 19 and 27 wells of PAM and IPKM cells inoculated with the 0.2% suspension in the second round of passage, respectively. In contrast, for sample 20901-1, HAD was confirmed in 2 and 7 wells of PAM and IPKM cells inoculated with the 2% suspension of the homogenate in the second round of passage, respectively, and no HAD reaction was detected in both cell cultures that were inoculated with the 0.2% suspension of homogenate. Regarding sample 201202, HAD reaction was confirmed in all wells of both cell cultures inoculated with the 2% suspension of homogenate and in 28 and 27 wells of PAM and IPKM cell cultures inoculated with the 0.2% suspension in the second round of passage, respectively.

### 3.3. Comparison of the Reproducibility of Virus Isolation in IPKM Versus PAM Cells

To verify the reproducibility of virus isolation between IPKM and PAM cells, 3 sets of IPKM cells and 3 batches of PAM cells were inoculated with newly prepared 2% sausage homogenates (Lot No.2). The overall isolation rates were decreased compared to the previous experiment, with 14, 12, and 13 wells of IPKM and 3, 9, and 4 wells of PAM cells being HAD-positive with homogenate 1-22. Likewise, 2, 4, and 5 wells of IPKM and 2, 1, and 1 wells of PAM cells were positive with the 201202 homogenate. All the wells inoculated with 1–21 and 20901-1 were negative (Table 3). The reduction of virus isolation rates in this experiment compared with the result shown in Table 2 might be due to heterogonous distribution of virus in the sausages, degradation by long-term storage, and freeze-thawing. 

### 3.4. Animal Experiment Using ASFV Isolates Obtained by IPKM Cell Cultures

**Figure 2:**
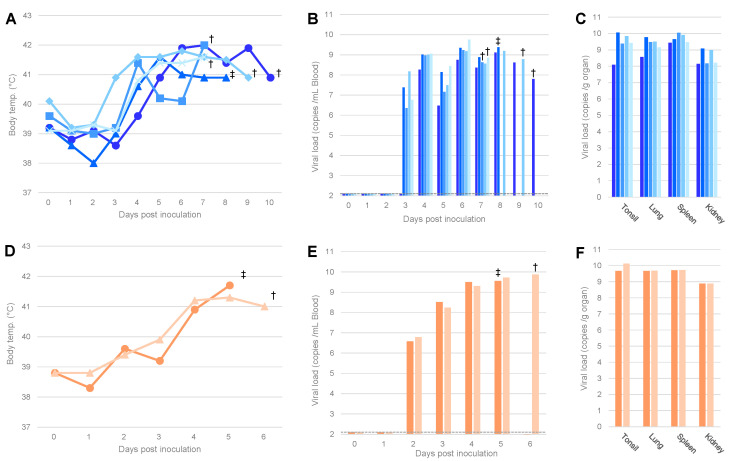
Body temperature and viral load of pigs inoculated with the isolated viruses. The body temperature (**A**,**D**) and viral copy numbers in the blood (**B**,**E**) and organs (**C**,**F**) of pigs inoculated with AQS-C-1-22 (blue, **A**–**C**) or AQS-P-20901-1 (orange, **D**–**F**) are shown. The identification numbers of pigs are indicated by the shape of markers or sorting order of bars: Nos. 1–5 are indicated by circles, triangles, squares, rhombi, and crosses (**A**) or ordered from left to right (**B**,**C**), respectively. Each symbol means euthanasia by humane endpoint (†) or death (‡), and the broken lines in B and E indicate the detection limit (=100 copies/mL of blood).

To assess the virulence of ASFVs isolated by IPKM cells, AQS-C-1-22 and AQS-P-20901-1 were inoculated into five and two pigs, respectively. All animals inoculated with the virus showed pyrexia (>40 °C) by 4 dpi (Figure 2A,D). AQS-C-1-22-inoculated pigs died at 8 dpi or were euthanized at 7, 9, and 10 dpi, whereas AQS-P-20901-1-inoculated ones died at 5 dpi or were euthanized at 6 dpi. The clinical symptoms of those pigs were high fever, anorexia, and severe depression that were similar to those observed in naturally infected pigs and in the pigs inoculated with the homogenates of the original samples, 1–22 and 20901-1, as described above (Figure 1).

As shown in Figure 2, ASFV-specific amplicons were detected in all tissue samples from infected animals, and their copy numbers were equally high (10^8^–10^10^ copies/g) compared with those of the pigs inoculated with the original pork products, as mentioned above (Figure 1).

### 3.5. Genomic Characterization of ASFV Isolates

**Figure 3:**
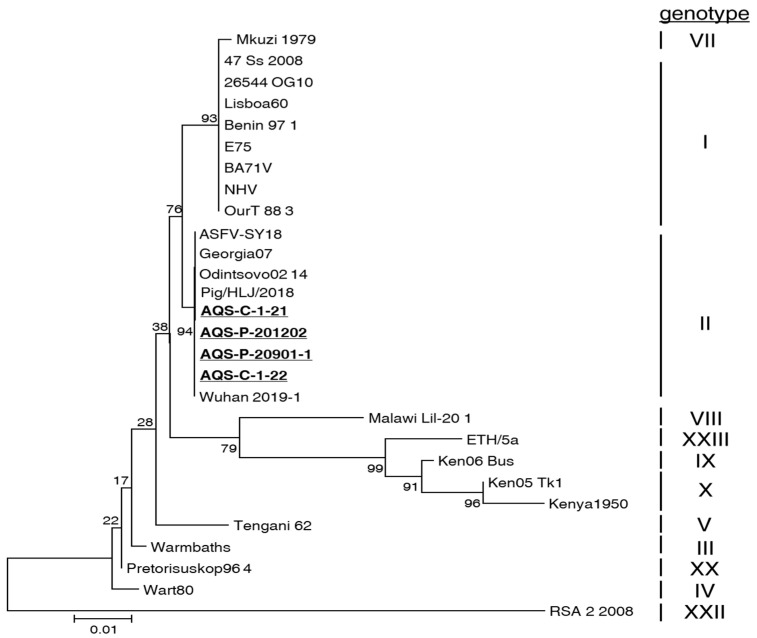
Phylogenetic tree of ASFV isolates generated based on the *B646L* gene sequence. Bootstrap values were calculated 1000 times and are expressed as percentages. The isolates obtained in this study are underlined.

To determine the genotype of the isolates obtained in this study, we performed a phylogenetic tree analysis of the nucleotide sequence data of the *B646L* gene (Figure 3). Based on the generated tree, all four isolates, i.e., AQS-C-1-21, AQS-C-1-22, AQS-P-20901-1, and AQS-P-201202, belonged to genotype II. Next, we analyzed the whole-genome sequencing data of all isolates. The data of the obtained short reads were mapped to the reference sequence of a Chinse strain of genotype II, Pig/HLJ/2018 (MK333180). All these isolates contained 183 open reading frames (ORFs) homologous to Pig/HLJ/2018. The whole-genome sequence identities between the isolates and the Pig/HLJ/2018 strain were more than 99%. The differences in the genomes between IPKM-isolated viruses and PAM-isolated ones after three rounds of passage were only a few amino acid substitutions caused by several nucleotide mutations (Table S1).

## 4. Discussion

In the present study, we compared infectivity and virulence between the viruses contained in pork products and those isolated by IPKM cell cultures. The pigs inoculated with the homogenate of the original material, 1–22, showed hyperthermia (>40 °C) and viremia that were similar to those of the pigs inoculated with the respective isolates, AQS-C-1-22. Similarly, the pigs inoculated with the homogenate of the original materials, 20901-1, showed pyrexia and viremia 4–6 days later than the pigs inoculated with the respective isolates, AQS-P-20901-1 (Figure 1D–F and Figure 2D–F). We suppose that the difference in disease onset between those materials may be at least partly attributed to the difference in virus titers in the inocula. In fact, the isolation success rate of the 2% homogenates prepared from pork products 1–22 and 20901-1 were 40/40 and 1/40 wells, respectively, in the first round of passage in IPKM and PAM cell cultures, indicating a difference in the infectivity of the viruses contained in those materials (Table 2). However, all pigs inoculated with homogenates or isolated viruses finally showed similar levels of virus loads in peripheral tissues and clinical signs, regardless of the inoculum (Figure 1 and Figure 2). This observation supports the hypothesis that virus isolation and maintenance in IPKM cell cultures do not change the biological features of ASFVs. We recently reported that IPKM was highly sensitive to ASFVs and applicable to the efficient propagation, titration, isolation, and genetic manipulation of the viruses without affecting the spontaneous mutation rate in the viral genome over several passages [10]. These characteristics suggest the suitability of IPKM cells for the diagnosis of ASF by isolating field pathogens from various specimens, such as the dead body of an animal, contaminated premises or environmental materials [15], and pork meat and other related products derived from infected animals, which might be circulating along with the value chain of pig production in epidemics. As an example, in this study, we attempted to isolate live viruses contained in PCR-positive pork products that were illegally imported for human consumption via international airports on different dates. By inoculating the samples into IPKM cells, we successfully detected and obtained multiple isolates of genotype II with similar efficiencies to authentic PAM cells. Moreover, comparison of virus isolation rates between IPKM and PAM cells showed that IPKM cells allow for more reliable and reproducible ASFV isolation than PAM cells (Table 3). In some cases, IPKM cells failed to support the growth of ASFVs in contaminated specimens (Table 2). These failures may be attributed to the presence of cytotoxic ingredients included in pork products, such as spices and preservatives, or to a high salt content in the testing samples, which may affect the viability or biological functions of IPKM cells. The present data indicate that the IPKM cells are presumably more sensitive to co-existing inhibitory materials than PAM cells.

Whole-genome sequencing data demonstrated that the viruses that were isolated in IPKM cell cultures contained only several nucleotide mutations compared with the PAM-passaged ones (Table S1). In this study, we used crude sausage homogenates, and it is possible that multiple viruses with mutations were mixed in the samples before isolation. Although previous studies have shown that viruses serially passed in IPKM cells rarely mutate [10], the possibility that mutations occurred during virus isolation cannot be ruled out. Further studies are needed to clarify which of the above is responsible for the mutations identified in this study.

Our present study indicates that IPKM cells support virus propagation without induction of genetic mutations and allow the stable growth of ASFVs in common laboratory settings also in the case of virus isolation. However, the deletions in the genome of several cell culture-adapted ASFVs were reported [16]. Therefore, genomic stability of ASFVs in IPKM cells is still an issue to be addressed.

## 5. Conclusions

The present study indicated that the IPKM cell line would be an ideal tool for the isolation of live ASFVs with high efficiency, comparable to that of PAM cells. The isolated viruses retained biologically identical characteristics compared with the original ones during the isolation and early rounds (at least less than three rounds) of passage *in vitro*.

## Figures and Tables

**Table 1:** Definition of the seized pork products used in this study.

Sample No.	Seized Item (Weight)	Departure	Destination	Reason for Discovery
1–21	Sausage (0.6 kg)	Shanghai	Nagoya	Customs inspection
1–22	Sausage (1.3 kg)	Qingdao	Nagoya	Oral interview
20901-1	Sausage (2.2 kg)	Manila	Tokyo (Haneda)	Detection dog
201202	Sausage (3.5 kg)	Manila	Tokyo (Haneda)	Customs inspection

**Table 2:** Number of wells showing HAD in PAM and IPKM cells inoculated with 2 and 0.2% sausage homogenates (Lot No.1).

		1–21		1–22		20901-1		201202	
		2%	0.2%	2%	0.2%	2%	0.2%	2%	0.2%
IPKM	1st culture	ND	ND	40	23	1	0	40	23
	2nd culture	5	0	40	27	7	0	40	27
	3rd culture	5	0	NT	27	7	0	NT	27
PAM	1st culture	19 *	3	40	17	1	0	40	19
	2nd culture	28	4	40	19	2	0	40	28
	3rd culture	28	4	NT	19	2	0	NT	28

*: Number of wells that showed HAD reaction/40 wells. ND: Not determined due to the cytotoxic activity of the sample. NT: Not tested.

**Table 3:** Comparison of the reproducibility of IPKM versus PAM cells for virus isolation using 2% sausage homogenates (Lot No.2).

		1–21	1–22	20901-1	201202
	Set 1	0 *	14	0	2
IPKM	Set 2	0	12	0	4
	Set 3	0	13	0	5
	Batch 1	0	3	0	2
PAM	Batch 2	0	9	0	1
	Batch 3	0	4	0	1

*: Number of wells that showed HAD reaction/40 wells.

## Data Availability

The genome information of our isolates was submitted to the GenBank: LC659086-LC659089.

## Supplementary Materials

The following supporting information can be downloaded at: https://www.mdpi.com/article/10.3390/v14081794/s1, Table S1: Genetic difference in the genome DNA of ASFV isolated in PAM and IPKM cells.
