# Supplementary material for: Usability of Immortalized Porcine Kidney Macrophage Cultures for the Isolation of ASFV without Affecting Virulence
Source: Viruses. 2022 Aug 16;14(8):1794. doi: 10.3390/v14081794 (PMC9414656; doi:10.3390/v14081794)
Supplement: Supplementary file 1 [file viruses-14-01794-s001.zip › viruses-1797680-supplementary.pdf]

Table S1. Genetic difference in the genome DNA of ASFV isolated in PAM and IPKM cells.

		AQS-C-1-21		AQS-C-1-22		AQS-P-20901-1		AQS-P-201202	
Gene	Position [a]	PAM	IPKM	PAM	IPKM	PAM	IPKM	PAM	IPKM
NCR [b]	426	CCCC	CCCC	C [c]	CCCC	CC	CC	CCCC	CCCC
MGF 110-14L	13265-70	AC	A	CCCCCCC	A	ACCCCC	ACCCCC	ACCCC	ACCCCC
MGF 110-13L	14708	CCCC	CCCC	CCCC	CCCC	- [d]	-	C	-
NCR	16664	GG	GG	-	GG	G	-	G	G
NCR	16877	G	G	-	G	-	-	-	-
NCR	19032-37	-----	-----	GGGGGG	-----	GGGGG	GGGGGG	GGGG	G
NCR	20835-37	---	---	GGG	---	GGGG	GGG	GGGGG	GGGGG
G1340L	110729	G	G	A	G	G	G	G	G

a: Nucleotide position number is based on the sequence of the ASFV Pig/HLJ/2018.
b: NCR is an abbreviation for non-coding region.
c: Under bars show the nucleotide differences between PAM and IPKM passaged viruses.
d: The bars indicate the nucleotide deletion compared with ASFV Pig/HLJ/2018.
